# A Breathtaking Case of Chemotherapy-Induced Pneumonitis

**DOI:** 10.7759/cureus.78791

**Published:** 2025-02-09

**Authors:** Anthony V Cook, David I LeRoy, Cassie T Konja

**Affiliations:** 1 Internal Medicine, Henry Ford Health System - Warren, Warren, USA

**Keywords:** chemotherapy-induced pulmonary toxicity, etoposide, etoposide-induced pneumonitis, germ cell tumor, metastatic seminoma

## Abstract

Medication-induced pulmonary toxicity is a rare adverse event that may occur with many chemotherapeutic agents, including etoposide. This agent has been found to cause various toxicities, including anaphylaxis, angioedema, hypotension, and pneumonitis. Etoposide is used in chemotherapy regimens for multiple cancers, including germ-cell tumors. Proper diagnosis of chemotherapy-induced pulmonary toxicity is imperative and should be considered in patients who develop acute respiratory failure during or after chemotherapy. Here, we discuss an unexpected case of etoposide-induced pneumonitis after a short course of IV etoposide in a patient with metastatic seminoma.

## Introduction

Chemotherapy-induced pulmonary toxicity is a growing concern, with symptoms ranging from cough and dyspnea to respiratory failure. Its diagnosis must be considered in patients who develop pulmonary symptoms during or after chemotherapy treatment. Etoposide is a DNA topoisomerase II inhibitor, which works on a molecular level to create breaks in DNA, ultimately leading to apoptosis. Etoposide is approved for single- and multi-agent regimens in the treatment of various neoplasms, including small-cell lung cancer, Kaposi’s sarcoma, and germ cell tumors [1]. Standard treatment for germ cell tumors in patients with a good prognosis typically involves three cycles of bleomycin, etoposide, and cisplatin, or four cycles of etoposide and cisplatin [2]. Unfortunately, etoposide is associated with multiple toxicities, most commonly including anaphylaxis, angioedema, chest discomfort, bronchospasm, and hypotension. In rare cases, etoposide has been associated with the development of pneumonitis and/or interstitial lung disease (ILD) [3,4]. Etoposide-induced pneumonitis can occur sporadically, with prophylactic corticosteroids showing no benefit [5]. Furthermore, literature suggests that etoposide-induced pneumonitis is more common with chronic oral etoposide use rather than with short courses of IV etoposide [4]. We present here a case of etoposide-induced pneumonitis after a short course of IV etoposide in a patient with metastatic seminoma. 

## Case presentation

A 36-year-old male with no significant past medical history was admitted with newly diagnosed, biopsy-proven stage III (T4N3M1a) good-risk right testicular seminoma with pulmonary metastasis. Initially presenting for testicular mass and pain, he was found to have right testicular seminoma with metastasis to the lungs via biopsy of the right lung mass. The biopsy stained positive markers for CD117 and placental alkaline phosphatase and negative for alpha fetoprotein, pankeratin, human chorionic gonadotropin (hCG), and CD30. Urology deemed the patient to be a poor surgical candidate. After a prolonged hospital stay complicated by a provoked right lower extremity deep vein thrombosis (DVT), he was scheduled to follow up with oncology for outpatient chemotherapy and was discharged home on apixaban. The patient was readmitted four days later after developing gross hematuria and severe anemia. Pertinent labs for both admissions are shown in Table 1. After hemoglobin stabilization, oncology recommended initiation of inpatient chemotherapy due to clinical deterioration. He was started on inpatient 216 mg IV etoposide and 43 mg cisplatin with 25 g mannitol with consideration for the addition of bleomycin in the outpatient setting. 

**Table 1:** Pertinent laboratory values from the first and second admissions INR: international normalised ratio, PTT: partial thromboplastin time, AFP: alpha-fetoprotein, HCG: human chorionic gonadotropin, LDH: lactate dehydrogenase, G6PD: glucose-6-phosphate dehydrogenase

Pertinent labs	First admission	Second admission	Reference range
WBC (K/mcL)	23.41	28.49	4.0-11.0
Hemoglobin (gm/dL)	9.6	4.2	13.5-17.5
Platelets (K/mcL)	743	1,133	150-400
Protime (seconds)	15.8	19.4	11.5-14.4
INR	1.28	1.63	N/A
PTT (seconds)	38.2	36.7	22.5-35.0
AFP (ng/mL)	<2.7	-	0-13.0
HCG (mIU/mL)	7	-	1.0-5.0
LDH (IUnits/L)	585	728	0-240
G6PD (minutes)	-	20	20-60
Absolute retiulocyte count (Mill/cu mm)	-	0.13	N/A
Reticulocyte (%)	-	3.70%	0.5-1.5
Haptoglobin (mg/dL)	-	415	30-200

**Figure 1:**
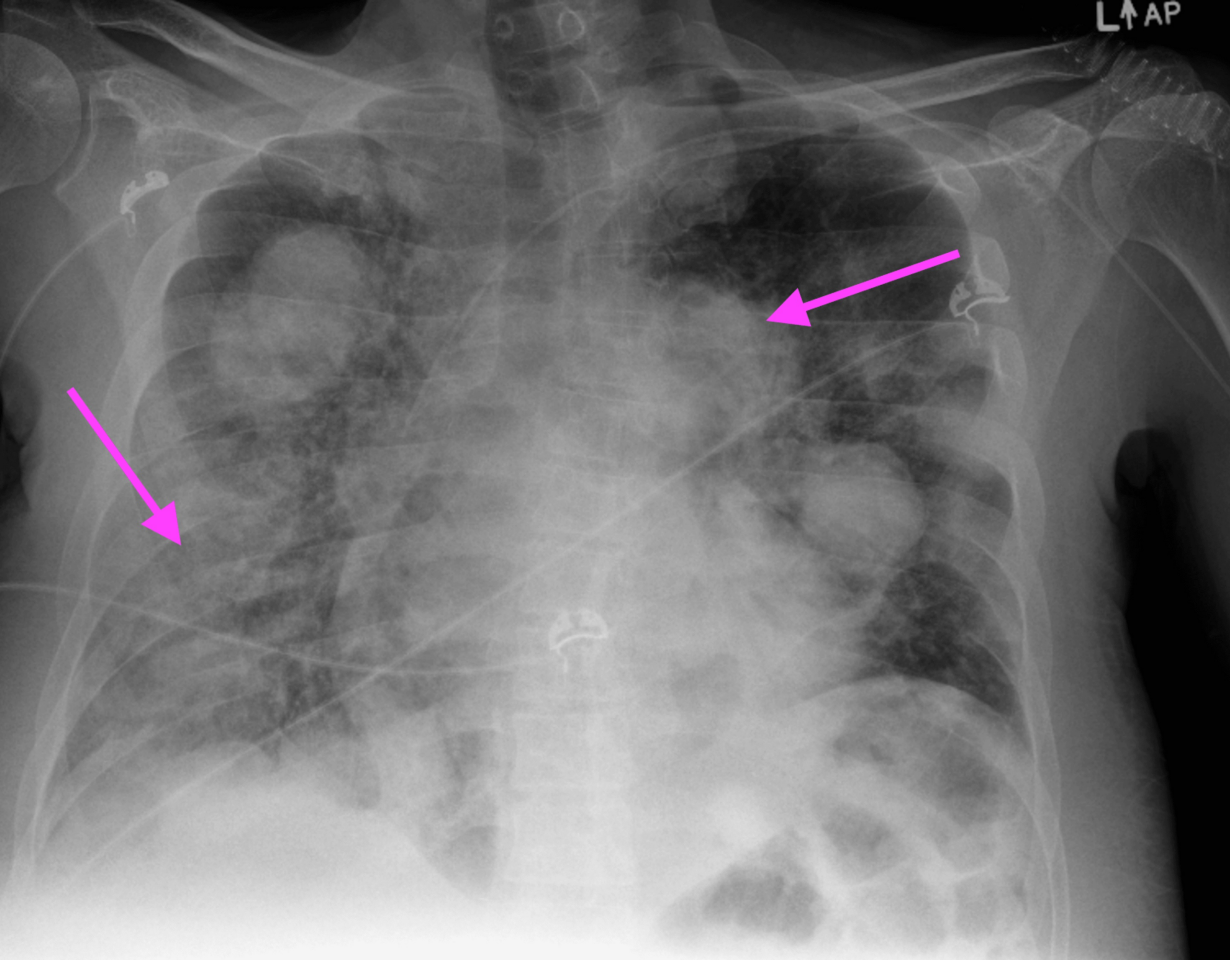
Chest X-ray showing new bilateral interstitial opacities after second dose of etoposide Chest X-ray showing new bilateral interstitial opacities while pulmonary metastasis remains unchanged, with arrows indicating the new areas of interstitial opacities.

**Figure 2:**
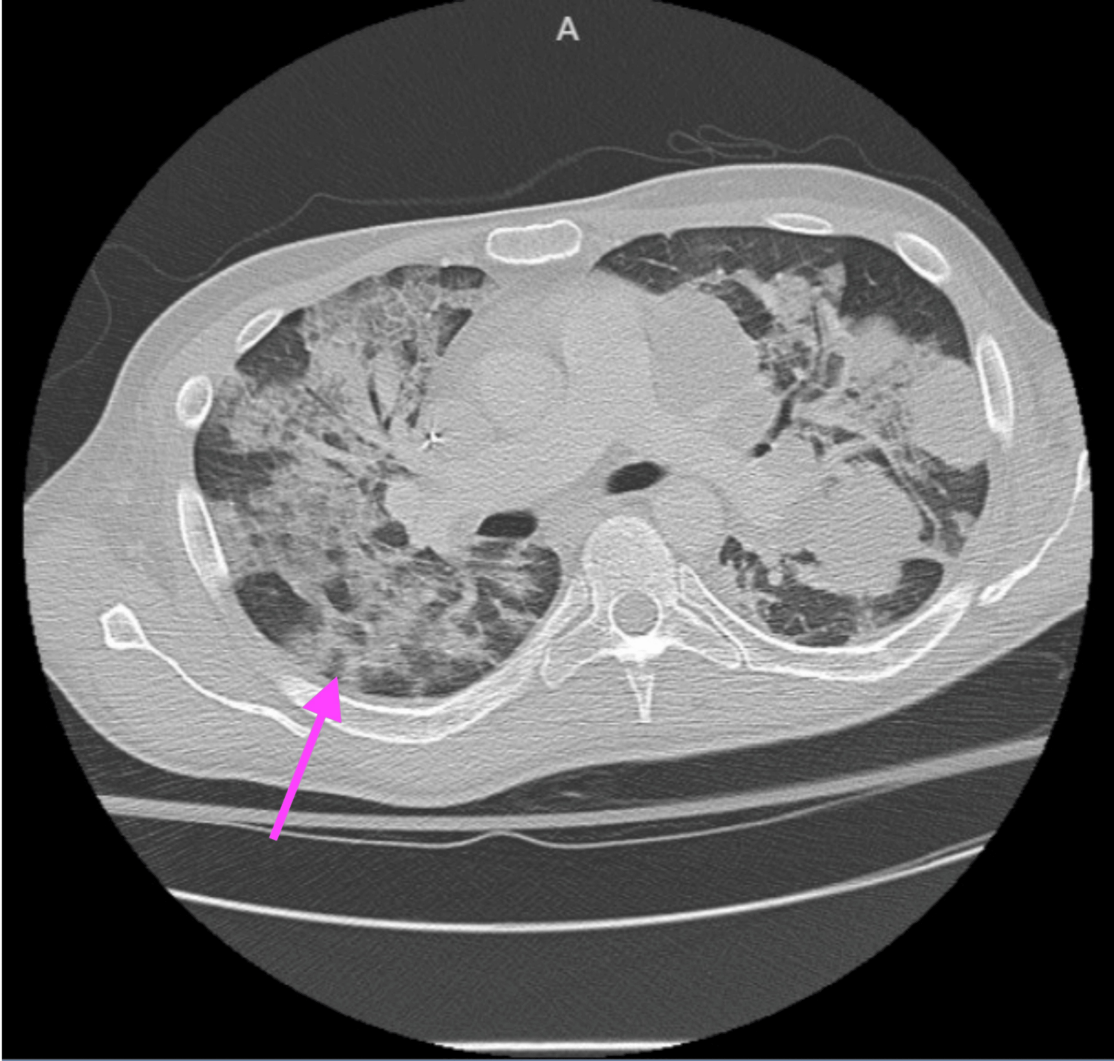
CT Angiography Chest showing new ground-glass opacities consistent with drug-induced pneumonitis CT angiography of the chest reveals unchanged pulmonary metastasis and new diffuse heterogeneous ground-glass opacities, with associated interlobular and intralobular septal thickening. An arrow highlights the area of new diffuse heterogeneous ground-glass opacities.

The day after receiving his second dose of etoposide, the patient’s oxygen saturation (SpO2) decreased to 86% on room air, improving to 93% after he was placed on a 15 L non-rebreather mask. Chest X-ray revealed new bilateral interstitial opacities, with unchanged pulmonary metastasis as seen in Figure 1. IV furosemide was given without improvement in his oxygen requirement. Echocardiogram showed a preserved ejection fraction without diastolic dysfunction or valvular abnormalities. CT angiography of the chest showed no pulmonary embolism, unchanged pulmonary metastasis, and new diffuse heterogeneous ground-glass opacities with associated interlobular and intralobular septal thickening demonstrated in Figure 2. The patient’s SpO2 then dropped to 85%, necessitating 60 L high-flow nasal cannula with fraction of inspired oxygen (FiO2) of 90%. An arterial blood gas (ABG) was ordered, revealing an arterial partial pressure of oxygen (pO2) of 60 mmHg, confirming significant hypoxemia requiring oxygen supplementation. The ABG, as well as, other pertinent lab results from the day after the second dose of etoposide are presented in Table 2. He was transferred to the step-down unit due to a high risk of respiratory collapse. Given the timing of his respiratory failure, the working diagnosis was drug-induced pneumonitis secondary to chemotherapy. Elective bronchoscopy with bronchoalveolar lavage and possible biopsy was offered to confirm the diagnosis; however, the patient refused and opted for empiric treatment.

**Table 2:** Pertinent laboratory values on the day of respiratory failure INR: international normalised ratio, PTT: partial thromboplastin time, AFP: alpha-fetoprotein, HCG: human chorionic gonadotropin, LDH: lactate dehydrogenase, pCO2: partial pressure of carbon dioxide, pO2: partial pressure of oxygen, HCO3: bicarbonate

Pertinent labs	Patient's Value	Reference range
WBC (K/mcL)	21.66	4.0-11.0
Hemoglobin (gm/dL)	10.1	13.5-17.5
Platelet (K/mcL)	532	150-400
Absolute Neutrophil (K/mcL)	19.58	1.8-7.5
Absolute Lymphocyte (K/mcL)	0.74	1.0-5.0
Protime (seconds)	13.9	11.5-14.4
INR	1.09	N/A
PTT (seconds)	29	22.5-35.0
LDH (IUnits/L)	585	0-240
AFP (ng/mL)	3.2	0-13
HCG (mIU/mL)	14	1.0-5.0
Lactic Acid (mmol/L)	2.5	0.5-2.0
Arterial pH	7.44	7.35-7.45
Arterial pCO2 (mmHg)	39	35-45
Arterial pO2 (mmHg)	60	75-100
Arterial HCO3 (mmol/L)	26	20-28

The patient was started on a seven-day course of empiric doxycycline and piperacillin-tazobactam, in addition to a three-day course of 250 mg IV methylprednisolone every six hours. His oxygen requirement decreased to 4 L via nasal cannula, and his chest X-ray showed resolution of interstitial opacities after completing the short course of high-dose steroids. The patient was transferred out of the step-down unit to a telemetry floor, where his oxygen requirement was further weaned to room air while on an oral steroid taper.

He was readmitted four days later for progressive dyspnea. During this hospitalization, he started the TIP regimen with 250 mg/m2 IV paclitaxel on day one of each cycle, 1,500 mg/m2 IV ifosfamide on days two to five of each cycle with mensa protection, and 25 mg/m2 IV cisplatin on days two to five of each cycle. He completed one cycle and, 20 days later, he started on Cycle 2, completing day one. On Cycle 2, day two, the patient only received ifosfamide, refusing cisplatin. Due to staffing issues and refusals, he skipped a day of therapy but completed Cycle 2, days three to five, receiving both ifosfamide and cisplatin, with an extra day for the missed cisplatin dose on day two. About a week after his last infusion, a rapid response was initiated due to difficulty breathing. He was found to be hypotensive, with a blood pressure of 78/37, and was placed on IV norepinephrine. Additionally, he was put on bilevel positive airway pressure (BiPAP) for hypoxia. He was transferred to the ICU, where he was intubated due to worsening hypoxia on BiPAP. The following morning, a CODE BLUE was called due to an initial rhythm of pulseless electrical activity, with no successful return of spontaneous circulation. 

## Discussion

Etoposide is a chemotherapeutic agent frequently used to treat germ cell tumors as part of a multi-agent regimen with either bleomycin, etoposide, and cisplatin, or etoposide and cisplatin [1,2]. Treatment with standard etoposide and cisplatin therapy, as with our patient, has shown a high response rate. One study involving etoposide and cisplatin in metastatic good-risk germ cell tumors demonstrated that 98% of the 289 patients achieved a complete response, with 269 of the 289 patients responding to chemotherapy alone [6]. Further studies demonstrated a three-year overall survival rate of 96% in patients with metastatic seminomas treated with etoposide and cisplatin [7]. Our patient was treated with a similar regimen of etoposide and cisplatin; however, unlike in these studies, he did not achieve resolution of his metastatic good-risk seminoma. The studies highlighted how uncommon it is for patients not to achieve a response, as only 17 patients failed to achieve a complete response in one study [6]. This regimen has demonstrated an excellent response with few documented side effects, the most common being neutropenia (48%) and neutropenic fever (12%) [7]. This underscores the rarity of adverse reactions, such as pulmonary toxicity.

While germ cell tumors often achieve complete responses with etoposide and cisplatin, the most reported side effect is neutropenia. Chemotherapy-induced lung toxicity is considered a severe reaction, with patients typically presenting with dyspnea, diffuse pulmonary infiltrates, and acute respiratory failure [8]. The risk of developing this toxicity often arises with second- or third-line agents, particularly in the context of lung cancer [8]. Etoposide has been rarely associated with pulmonary toxicities. Etoposide-induced pneumonitis has been described in prior literature, particularly with chronic use of oral etoposide therapy [9]. One case being reported in Japan detailed a patient who developed etoposide-induced pneumonitis after seven months of treatment [10]. Tissue examination via bronchoalveolar lavage has shown alveolar edema and damage with atypical type II pneumocytes; however, obtaining tissue samples can be challenging, as background disease, severe drug/radiation-induced ILD, or infection can be difficult to distinguish from drug-induced diffuse alveolar damage [8,9]. Additionally, some patients may be too ill to undergo such procedures [9]. Current treatment for etoposide-induced pulmonary toxicity includes cessation of etoposide and initiation of steroids, though results with this strategy have been variable, with better outcomes noted for patients with more acute or recent onset [8,9].

Although we were unable to confirm the diagnosis with pathology, drug-induced pneumonitis secondary to etoposide remains the most likely cause of this patient’s respiratory failure. Diffuse alveolar hemorrhage was considered; however, the chest X-ray findings did not correlate with the typical presentation of this condition. Occult bacterial and/or fungal pneumonia was also a consideration, given the patient’s newly immunocompromised status; however, this was unlikely due to a lack of systemic inflammatory response. Discontinuation of etoposide and initiation of steroids likely had a more significant impact on resolution of hypoxia than initiation of antibiotics, as oxygen demand escalated despite early antibiotic administration, with the patient returning to baseline oxygen status only after the steroid taper. It is undeniable that bronchoscopy with bronchoalveolar lavage would have assisted with definitively ruling out an infectious etiology, had the patient been agreeable. Nevertheless, the patient’s improved clinical course suggests the appropriate treatment was initiated. If left untreated, etoposide-induced pneumonitis carries the risk of developing into ILD, especially in the setting of concurrent methotrexate and/or thoracic radiation administration [3]. A high index of clinical suspicion is needed to prevent these adverse outcomes.

## Conclusions

Chemotherapy-induced pulmonary toxicity can range from milder symptoms, such as cough and dyspnea, to more serious concerns like respiratory failure. Various chemotherapeutic agents can cause pulmonary toxicity, including pneumonitis and ILD, with rare cases of etoposide-induced pneumonitis. Etoposide-induced pneumonitis has been more associated with prolonged courses of oral therapy; rarely has this syndrome been described in the context of short-course IV treatment. Etoposide has been shown to have a relatively safe side effect profile in the treatment of germ cell tumors, with most of the patients achieving complete response and only a few experiencing more severe reactions. While bronchoalveolar lavage is often performed for definitive diagnosis, our patient opted for empiric treatment, refusing biopsy or drug rechallenge. Given his improvement following the cessation of etoposide and the administration of steroids, etoposide-induced toxicity remains the leading diagnosis. If suspicion for etoposide-induced pneumonitis is high in the acute setting, treatment with steroids should commence until symptoms resolve to prevent the development of ILD, as failure to treat this syndrome appropriately can result in ILD.
